# Comparative effect of electroacupuncture with different frequency on headache attacks in migraine outpatients: study protocol for a randomised placebo-controlled trial

**DOI:** 10.1186/s13063-021-05429-9

**Published:** 2021-07-23

**Authors:** Na Nie, Le Chen, Tong Li, Chuanlong Zhou, Bangwei Li, Conghua Ji, Jie Zhou, Qin Chen, Qiushuang Li, Yi Liang, Jianqiao Fang

**Affiliations:** 1 Department of Acupuncture, The Third Affiliated Hospital of Zhejiang Chinese Medical University, No. 219 Moganshan Road, Xihu District, Hangzhou, 310005 Zhejiang Province China; 2 The Third School of Clinical Medical, Zhejiang Chinese Medical University, No. 548 Binwen Road, Binjiang District, Hangzhou, 310053 Zhejiang Province China; 3 The Clinical Research Institute of Zhejiang Provincial Hospital of TCM, No. 54 Youdian Road, Xihu District, Hangzhou, 310006 Zhejiang Province China

**Keywords:** Study protocol, EA, Migraine, Randomised placebo-controlled trial

## Abstract

**Background:**

Headache attacks severely impaired life quality and increase the economic burden of migraineurs. Electroacupuncture (EA) has been used worldwidely to treat several pain-related diseases including migraines. However, whether EA with low or high frequency exerts a distinct analgesic effect remains unknown and needs further study.

**Methods/Design:**

This study is a randomised, single-blinded, placebo-controlled trial with three parallel arms. A total of 144 migraine outpatients will be randomly allocated to the 2 Hz EA group, 100 Hz EA group and placebo control group. The duration of the trial is 20 weeks, including a 4-week-long baseline assessment period (weeks − 4–0), a 4-week-long treatment period (weeks 1–4) and a 12-week-long follow-up period (weeks 5–16). Twelve treatment sessions will be performed over a 4-week period (weeks 1–4). The primary outcome will be measured by the frequency of migraine attacks in the past 4 weeks at the end of week 4 post-randomisation. The secondary outcome will be measured by the frequency of migraine attacks in the past 4 weeks at the end of weeks 8, 12 and16 post-randomisation; number of days with migraine; dosage of ibuprofen; the scores of visual analogue scale (VAS); Self-Rating Anxiety Scale (SAS); Self-Rating Depression Scale (SDS); and Migraine Specific Quality of Life questionnaire (MSQ) in the past 4 weeks at the end of weeks 4, 8, 12 and 16 post-randomisation. Safety assessment, compliance and blinding evaluation will be carried out at the end of week 16 post-randomisation.

**Discussion:**

The recruitment will be started on 1 June 2021 and expected to finish on 31 May 2023. We aimed to clarify the dominant frequency of EA on headache attacks in a migraineur.

**Trial registration:**

Chinese Clinical Trial Registry ChiCTR-1800017259. Registered on 20 July 2018.

**Supplementary Information:**

The online version contains supplementary material available at 10.1186/s13063-021-05429-9.

## Background

Migraine is a common and frequent disorder in the department of neurology [1, 2]. It occurs on one side or both sides onset of pulsatile headache which can be accompanied by prodromal symptoms such as nausea, vomiting, photophobia, phobia, depression and burnout. Migraine has a long duration, frequent recurrence, difficulty in healing and even causes severe disability. The World Health Organization (WHO) Report listed migraine was the 19th major cause of disability for many years, and the disability caused by severe migraine was regarded as quadriplegia [3–5]. An epidemic survey investigated in the mainland of China showed the yearly prevalence of migraine was 9.3% (male, 5.9%; female, 12.8%) which was close to the global average of 11% [6]. Epidemiological studies in 2016 demonstrated that the global age-standardised prevalence of migraine was 14.4% in all which was 18.9% for women and 9.8% for men [7]. More than half of migraineurs reported the quality of daily work and life was affected [1, 2, 8]. Migraineurs suffered from a substantial decline in productive capacity, which led to increasing economic burden and low-quality of life [9]. It had been reported that the total annual direct costs for migraine in China were CNY 58.0 billion (USD 8.4 billion), and the total annual indirect financial losses to society due to migraine-induced lost productivity were CNY 273.7 billion (USD 39.4 billion) [6]. Migraine became the third cause of disability in people under 50 years of age according to the Global Burden of Disease Study 2015 (GBD 2015) [10]. Migraine not only impaired human health, but also aggravated health-related economic burden which further imposed a negative impact on quality of life [6, 9, 11, 12]. Thus, how to effectively prevent and control migraine attacks has been a hot area of research, which attract an increasing attention of clinical researchers all over the world. Up to now, the mechanisms of migraine attack are not fully understood, and the lack of migraine-targeted drugs still bother migraineurs. Drugs such as beta-adrenoceptor blockers and antidepressants are usually prescribed to alleviate headache attacks. However, medication-induced side effects limited clinical effectiveness and reduced patient-based compliance [11, 13].

Electroacupuncture (EA) is one of the most extensively applied acupuncture therapy and has been used worldwide for several decades in treating chronic pain. Accumulating evidences had been demonstrated that EA has a good analgesic effect for a variety of pain, including inflammatory pain and neuropathy pain [14–16]. It was generally known that the analgesic effect of 2 Hz EA is superior to 100 Hz EA for neuropathic pain [17, 18]. Cochrane systematic reviews indicated that acupuncture or EA has a definite curative effect and the advantage for safety on treating migraine [19–21]. Zhao et al. further demonstrated true acupuncture has a long-term reduction in migraine recurrence long-term effect of acupuncture for migraine [22]. However, the therapeutic difference between low-frequency EA and high-frequency EA on migraine remains unknown. Therefore, the study is designed to compare the analgesic effect of EA with different frequencies, further provide more evidence to select the optimal frequency of EA on migraine.

## Methods/design

### Study design

**Fig. 1:**
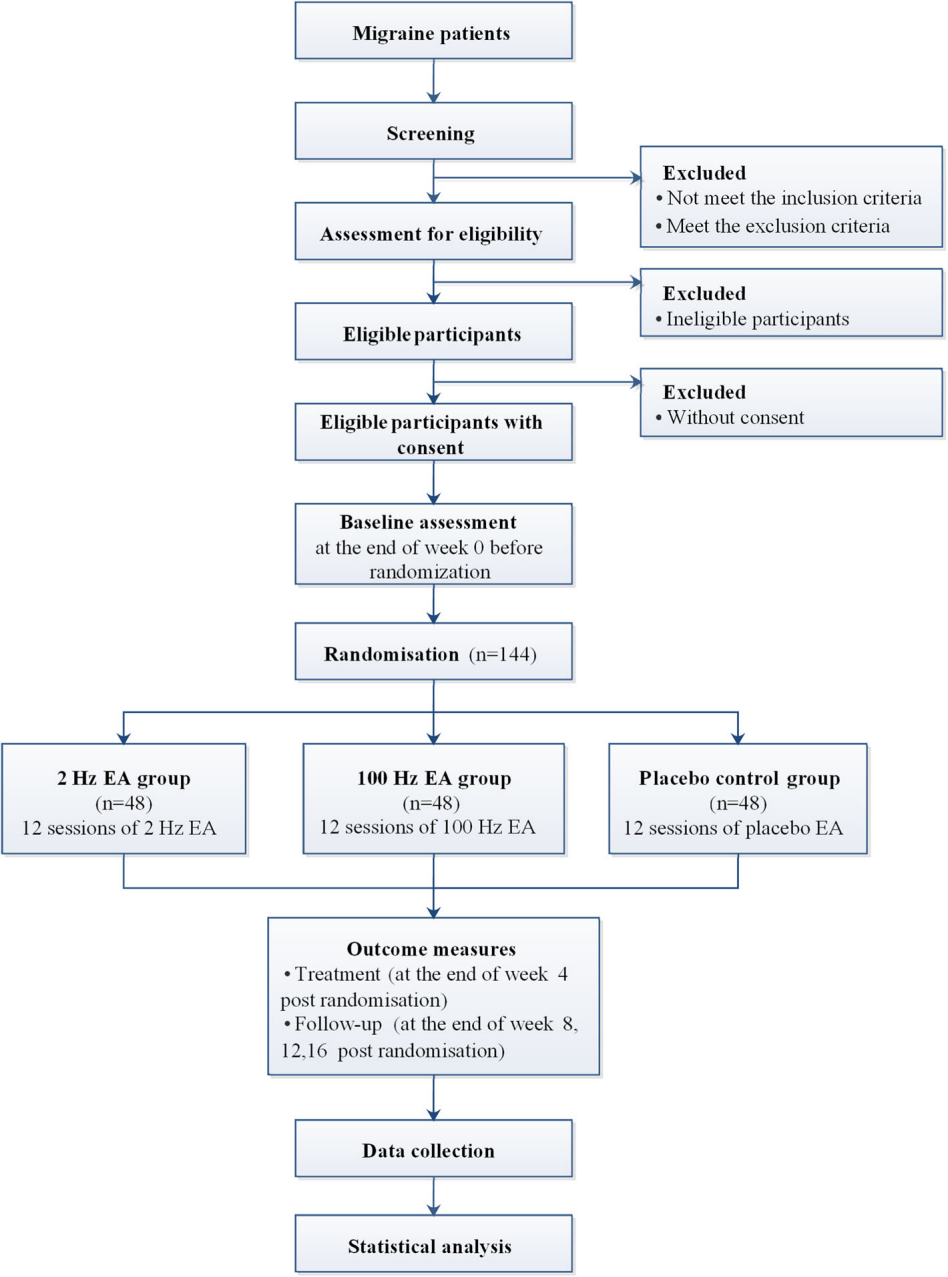
Flow diagram of study design

The study is a randomised, single-blinded, placebo-controlled clinical trial with three parallel arms. It aims to clarify the therapeutic effect of EA with low or high frequency on headache attacks in migraineurs and to screen out the dominant frequency of EA. Participants will be randomised to the 2 Hz EA group, the 100 Hz EA group and the placebo control group (1:1:1 ratio). After the baseline assessment has been carried out and the informed consent has been obtained, a randomisation number will be sent to the acupuncturist by phone or message. The statisticians, outcome assessors and neurologists will be blinded to the allocation. The flowchart of the trial is presented in Fig. 1. The duration of the trial is 20 weeks, including a 4-week-long baseline assessment period (weeks − 4–0), a 4-week-long treatment period (weeks 1–4) and a 12-week-long follow-up period (weeks 5–16). The EA group will receive 12 sessions of EA stimulation in total for 4 weeks, and the placebo control group will receive sham EA with superficial insertion of needles into sham acupoints and without electrical stimulation. And we will formulate the project to improve compliance by establishing good communication relationships via telephone calls during the 16 weeks of treatment and follow-up periods.

The trial was obtained the registration number ChiCTR-1800017259 in the Chinese Clinical Trial Registry (www.chictr.org.cn).

### Participant recruitment

Patients will be recruited in the Third Affiliated Hospital of Zhejiang Chinese Medicine University in Zhejiang province, China. We will propagate by posters in the hospital and the Internet. Potential participants will be scheduled to visit the hospital by calling from the researchers’ telephone. Assessment for eligibility will be carried out by neurologists at the 1st visit, and the result will respond to patients immediately. Eligible patients will be explained more details of this trial, and they have 1 week to decide to participate or not. During this 1-week period, they will have an opportunity to ask questions about this trial. Once potential participants agree to participate, they will be scheduled for the 2nd visit to the hospital and signed the written consent. Participants will be giving a full explanation of the purpose, nature and potential risks of the trial and informed that they have the right to withdraw from the trial at any time. Baseline assessment and random allocation will be done during the 2nd visit. All participants were reimbursed with transportation fees and free 12-session EA treatment.

Participants will not take any regular medications for migraine, but only be allowed to take an ibuprofen extended-release capsule once when necessary (0.3 g per capsule, Sino-American Schweppes Co., Ltd., Tianjin City, China) which must be recorded, and the frequency must be recorded in the past 4 weeks at the end of weeks 4, 8, 12 and 16 post-randomisation.

### Inclusion criteria

All collected migraineurs will be diagnosed as migraine with or without aura by neurologists with the criteria of the International Headache Classification (3rd edition, ICHD-III), which were set by the International Headache Society (IHS) [23]. Participants must also match the following criteria: (1) those aged 18–65 years with more than 1 year of migraine history; (2) have had 2–8 headache attacks, but less than 15 days of attacks per month during the previous 3 months and during baseline measurement; (3) have completed the headache diary of self-reported baseline assessment; and (4) written informed consent can be provided by themselves or guardians when participants are illiterate or semi-illiterate.

### Exclusion criteria

Participants meeting any of the following criteria will be excluded: (1) headache resulted from craniocerebral disease such as cerebral vascular diseases, intracranial aneurysms, space-occupying lesions and other intracranial organic diseases; (2) headache caused by cluster headache, hemiplegic, basilar migraine, medication overuse or tension headache; (3) have taken prophylactic medication (e.g. beta-adrenoceptor blockers, calcium antagonists, antiepileptic drugs, antidepressant drugs, 5-HT receptor blockers) or received acupuncture treatment in the previous 1 month; (4) have a mental illness or a serious cognitive impairment; (5) suffer from any serious disease of cardiovascular, cerebrovascular, hepatic, renal and haematopoietic systems; (6) those who are pregnant or lactating or planning to become pregnant; (7) those who had participated in the other clinical trials at the same time; and (8) those who had the structural and vascular lesions of the brain and blood vessel measured by cerebral CT imaging and CT angiogram or cerebral MRI imaging and MR angiogram.

### Ethical considerations

The protocol of this study was approved by the Medical Ethics Committee of the Third Affiliated Hospital of Zhejiang Chinese University (permission number: ZSLL-KY-2017-036).

### Randomisation and blinding

The randomisation scheme of the study will be generated by the Zhejiang Provincial Clinical Research Institute of TCM and eligible participants will be randomised into the 2 Hz EA group, 100 Hz EA group or placebo control group. The researcher will contact the research institute after the baseline visit and the institute then passes on the allocation to the acupuncturist for treating the participant. All participants and study researchers including outcome assessors and statisticians will be blinded to the group assignments. However, it is impossible to blind the acupuncturists who would not allow to take part in any evaluation and recruitment process. Acupuncture expectancy assessment will be operated before randomisation (week 0) and blinding evaluation will be carried out at the end of this trial (week 16 post-randomisation).

After the data analysis, the unblinding will be carried out. In case of emergency or the subject needs to be rescued, the researcher will disassemble it according to the procedures in the protocol. Once disassembled, the numbered case will be discontinuing, and the reason for suspension of treatment will be recorded in the case report form. Outcome data for discontinued cases will still be recorded during follow-up.

### Interventions

All participants will receive EA treatment or placebo control treatment in the acupuncture clinic of the Third Affiliated Hospital of Zhejiang Chinese Medicine University. Participants will be randomised to receive different treatments during weeks 1–4 post-randomisation. Participants in the two EA groups will receive 2 Hz or 100 Hz EA stimulation, while those in the placebo control group will receive superficial acupuncture in sham points with sham EA stimulation. Senior acupuncturists with more than 5 years of acupuncture clinical experience will be allowed to participate in the trial at the completion of training including therapeutic regimen and details of different EA and placebo EA manipulation. The location and specific operation of the EA groups and the placebo control group are shown in Table 1.

**Table 1:** Details of the EA group and placebo control group

Group	Acupoint	Manipulation
**EA group (2 Hz and 100 Hz)**	(i) *Fengchi (GB20)*	(i) Is punctured obliquely 15–30 mm toward to the apex nasi
	(ii) *Gongxue (extra-point)*	(ii) Is punctured obliquely 15–30 mm toward to the apex nasi
	(iii) *Shuaigu (GB8)*	(iii) Is punctured horizontally 10–20 mm backward
	(iv) *Taiyang (EX-HN5)*	(iv) Is punctured obliquely 7.5–15 mm backward
	(v) *Sizhukong (SJ23)*	(v) Is punctured obliquely 10–20 mm backward
	(vi) *Waiguan (SJ5)*	(vi) Is punctured perpendicularly 10–20 mm
	(vii) *Yanglingquan(GB34)*	(vii) Is punctured perpendicularly 15–30 mm
**Placebo control group**	(i) *Fengchi (GB20)* outward 1 cm by side	(i) Is punctured obliquely 7.5–15 mm toward to the apex nasi
	(ii) *Gongxue (extra-point)* outward 1 cm by side	(ii) Is punctured obliquely 7.5–15 mm toward to the apex nasi
	(iii) *Shuaigu (GB8)* backward 1 cm by side	(iii) Is punctured horizontally 5–7.5 mm backward
	(iv) *Taiyang (EX-HN5)* backward 1 cm by side	(iv) Is punctured obliquely 5–7.5 mm backward
	(v) *Sizhukong (SJ23)* backward 1 cm by side	(v) Is punctured obliquely 5–7.5 mm backward
	(vi) *Waiguan (SJ5)* outward 1 cm by side	(vi) Is punctured perpendicularly 5–10 mm
	(vii) *Yanglingquan (GB34)* outward 1 cm by side	(vii) Is punctured perpendicularly 7.5–15 mm

#### 2 Hz EA group

The acupoint prescription (Table 1) is designed by an experienced acupuncturist who spend more than 30 years practising electroacupuncture analgesia. According to TCM’s theory, migraine attacks always happened at the head’s unilateral temporal side which belonged to the Shaoyang meridian according to TCM; thus, the acupoints were selected from and near the Shaoyang meridian. The local skin of all acupoints should be disinfected when patients are in a comfortable sitting position. Sterile needles (0.25 mm in diameter, 40 mm in length, Suzhou Medical Appliance in Suzhou, Jiangsu Province, China) will be inserted into each acupoint, and the depths will be adjusted by the acupuncturist until the arrival of *deqi* sensation. Then *Fengchi (GB20)* and *Gongxue (extra-point)* in homolateral will be connected to the output terminals of the HANS Acupuncture point Nerve Stimulators (HANS-200A, Nanjing Jisheng Medical Technology Co., Ltd., Nanjing, Jiangsu Province, China) and stimulated with low-frequency (2 Hz) EA and appropriate endurable intensity. Participants will be given one session per day for 30 min, 3 times per week (every other day recommended) and 12 sessions/4 weeks in total.

#### 100 Hz EA group

The EA frequency will be set to 100 Hz, but beyond that, both acupoint selection and specific operation will be the same as the 2 Hz EA group.

#### Placebo control group

Sham points will be selected nearby the real acupoints, and the locations are shown in Table 1. Needles will be inserted superficially to sham points needling without the arrival of *Qi*. That means the meridians and other acupoints should be avoided. Meanwhile, sham EA (the output circuit of the apparatus will be cut-off) will be operated during the trial period as the same as real EA besides electrical stimulation.

### Assessments

At the last week of each time frame, participants will be scheduled to visit the acupuncture clinic of the hospital. Assessment will be carried out by independent assessors blinded to the allocation. Detailed time points of outcome measures are provided in Table 2.

**Table 2:** Timetable of treatment and outcome collection

Period	Screening	Baseline	Treatment	Follow-up		
Visit	1	2	3	4	5	6
**Time frame**	/	Weeks − 4–0	Weeks 1–4	Weeks 5–8	Weeks 9–12	Weeks 13–16
**Inclusion/exclusion criteria**	×					
**Demographic characteristics**	×					
**Informed consent**		×				
**Random allocation**		×				
**EA/placebo control treatment**			×			
**Frequency of migraine attacks**		×	×	×	×	×
**Number of days with migraine**		×	×	×	×	×
**Dosage of ibuprofen**		×	×	×	×	×
**Visual analogue scale (VAS)**		×	×	×	×	×
**Self-Rating Anxiety Scale (SAS)**		×	×	×	×	×
**Self-Rating Depression Scale (SDS)**		×	×	×	×	×
**Migraine-Specific Quality of Life Questionnaire (MSQ)**		×	×	×	×	×
**Acupuncture expectancy assessment**		×				
**Safety assessment**						×
**Compliance evaluation**						×
**Blinding evaluation**						×

#### Baseline assessments

Baseline assessments will be conducted before randomisation, including gender, age, height, weight, course of migraine, family history of migraine (yes or no), frequency of migraine, number of days with migraine, dosage of ibuprofen, the scores of VAS, SAS, SDS and MSQ in 4 weeks and acupuncture expectancy assessment. The assessments include participants whether they had received EA treatment and expectations of EA treatment for patients.

#### Primary outcome assessment

The primary outcome assessment will be a frequency of migraine attacks during the treatment period (weeks 1–4) between the 2 Hz and 100 Hz EA groups, which will be recorded at the end of week 4 post-randomisation. The frequency of migraine attacks means the number of headache attacks per 4 weeks which calculated according to the Guidelines for Controlled Trials of Migraine Drugs (3rd edition) published by the International Headache Society in 2012 [24].

#### Secondary outcome assessments

The secondary outcomes will be evaluated per 4 weeks with the frequency of migraine attacks during the treatment period (weeks 1–4) between each EA group and placebo control group, those during the follow-up periods (weeks 5–16) among the three groups. In addition, secondary outcomes will be evaluated with the number of days with migraine, dosage of ibuprofen and the scores of VAS, SAS, SDS and MSQ, which will be recorded per 4 weeks at the end of weeks 4, 8, 12 and 16 post-randomisation (Table 2). Participants will be recorded by the closest score for the most painful condition of migraine attacks per 4 weeks. The total scores of the Self-Rating Anxiety Scale (SAS), Self-Rating Depression Scale (SDS) and Migraine Specific Quality of Life questionnaire (MSQ) will be respectively reflected anxiety status, depression status and actual life state.

### Incidence of adverse events

Any adverse event (AE) information will be got by participants’ report and determined by the acupuncturists at treatment and follow-up periods. Acupuncturists will be responsible for the recording of the EA-related AES on the case report form (CRF). The AEs and how they are dealt with will be recorded during the 4-week treatment and three consecutive 4-week follow-ups. AEs including bleeding, hematoma, broken needle, fainting, severe pain and local infection may occur by needing. The acupuncturists also will decide whether the adverse events are serious adverse event (SAE). Any SAE-like severe local infection can lead to driving the disease process. However, EA treatment is not usually associated with SAEs. If participants suffer AEs or SAEs, all details such as the date of occurrence, time, degree, measurement related to the treatment, course, outcome and causal relationship with the treatment will be documented. Moreover, if SAEs occur in participants, physicians should immediately provide the appropriate care to ensure their safety. Additionally, SAEs will be reported to the principal investigator immediately and also to the clinical centre and the medical ethics committee in 24 h.

### Quality control

The protocol has been perfected by experienced acupuncturists and statisticians before the trial. In addition, all researchers are required to take part in a series of training sessions including research protocol, standard operating procedures and acupuncture practical lessons. There will be arranged monitor to supervise the operators and discuss the progress every 3 months during the trial. Data input and storage will be performed by the Zhejiang Provincial Clinical Research Institute of TCM. And we will input the data of double entry for ensuring the accuracy. The principal investigator and the statistician will have access to all data in the research. If serious safety problems occur in the trial, such as serious adverse events or serious complications, the trial will be terminated. The principal investigator will make the final decision on the termination. It will be not necessary to break the blind when the anticipated SAEs occur.

### Sample size calculation

The sample size of this study is estimated by the method of two samples mean comparison. A high-quality randomised controlled trial by EA of single frequency for migraine has not been reported. For this reason, we do not use data from the literature to calculate the sample size. Based on a previous pilot test, the frequency of migraine attacks at the treatment period of 2 Hz was 1.11 and 100 Hz EA group 2.13. The standard deviation of 2 Hz EA was 1.49 and 100 Hz EA group 1.75. *σ*^2^ and *δ*^2^ equalled to 2.64 and 1.14, respectively, according to the above data. For this trial, it has been determined prospectively that *α* = 0.05 and 1-*β* = 0.80, then we looked up table that *Z*_*α*_ = 1.96 and *Z*_*β*_ = 0.84. The sample size will be calculated by the following equation.

$$ n=\frac{{\left({Z}_{\alpha }+{Z}_{\beta}\right)}^2\ast 2{\sigma}^2}{\delta^2} $$

Thus, at least 40 participants are required for each group which is counted by the SAS 9.1.3 (SAS Institute, Cary, NC, USA) software. To compensate for a prevalence of withdrawal of 20%, we plan to recruit 96 participants in the two EA groups, with 48 participants for each group.

The effectiveness of EA in treating migraine has been confirmed in some literatures. And in order to further confirm the efficacy of EA in treating migraine, we added a placebo control group with 48 participants to a 1:1:1 ratio. Then, the sample size of the three groups was required 144 participants in total.

### Statistical analysis

We will express the results of the statistical analysis with the principle of modified intention-to-treat (MITT), which will be defined as all the participants who have baseline data. All patients who received at least one session of their assigned treatment will be included in the safety analyses. MITT analysis will be performed to analyse all randomised data, and the last observation carried forward (LOCF) will be adopted to impute the missing values.

Baseline characteristics will be shown according to the study groups as means (SD) or median (IQR) for continuous variables and as numbers and percentages for categorical variables. The primary outcome will be described utilising the mean (standard deviation) when following a normality assumption and using the median (quartile spacing) when the normality assumption is violated. For the primary outcome, the difference between the frequency of migraine attacks during the treatment period (weeks 1–4) in the 2 Hz EA group and 100 Hz EA group will be assessed with an analysis of covariance (ANCOVA) model, which controlled by baseline frequency of migraine attacks reported during the 4 weeks prior to randomisation (week − 4–0). For the secondary outcome, continuous variables with repeated measurement, including frequency of migraine attacks in the 2 Hz EA group and 100 Hz EA group respectively compared to the placebo control group during the treatment period (weeks 1–4), frequency of migraine attacks during follow-up periods (weeks 5–16), days with migraine, dosage of ibuprofen, the scores of VAS, SAS, SDS and MSQ during treatment periods (weeks 1–4) and three follow-up periods (weeks 5–16) will be evaluated by a mixed-effect model with repeated measurement methods. One-way analysis of variance (ANOVA) or Kruskal-Wallis one-way ANOVA (if normality is violated) will be used for comparison among the three groups. The analysis results will be considered to indicate statistical significance when *P* < 0.05.

Statistical analyses will be done by using R (version 4.0.0) and Stata 16/SE. All tests of significance were two-tailed, and a *P* value of 0.05 or less was considered to indicate statistical significance for the primary end point. Because of the potential for type I error due to multiple comparisons, findings for analyses of secondary end points should be interpreted as exploratory.

## Discussion

Acupuncture, as the vital part of traditional Chinese medicine, has been becoming more and more popular all over the world. Moreover, acupuncture has been registered and accredited in many countries. EA is a novel type of acupuncture and becomes more acceptable than traditional acupuncture due to its stronger analgesic potency and parameter-adjustable features.

Previous systematic reviews and clinical trials had indicated that acupuncture and EA exerted a favourable analgesic effect on migraine [19–22]. Previous studies showed that the underlying mechanism between low-frequency and high-frequency EA had distinct, low-frequency EA release enkephalin and endorphin which can last for a long time whereas high-frequency EA release dynorphin rapidly which get the analgesic effect immediately [16]. According to professor Han’s achievements in scientific research, 2 Hz EA had been recommended to treat neuropathic pain [16]. For the lack of trials of EA with different frequencies for migraine, the frequency preference of EA for treating migraine remains uncertain. Therefore, we designed a parallel-design, patient-assessor blind, RCT to explore the dominant frequency of EA on migraine. According to the Guidelines for controlled trials of drugs in migraine [24], it has been recommended that either 4 weeks or 1 month is considered an appropriate evaluation interval. Studies had shown that negative emotion has a close relationship with pain diseases and changes in pain-related affection had been improved by acupuncture [25–28]. In this trial, psychological and emotional condition of participants will be assessed by using SAS and SDS scale. Migraine Specific Quality of Life questionnaire (MSQ) is the specific evaluation method more widely used for quality of life in migraine patients. As pain influences the life quality of the patients, we designed MSQ for the evaluation of quality of life [6, 11, 12].

The placebo control group was operated by superficial needling for non-acupoint and sham EA in this trial. Although all steps of sham EA manipulation are equal to real EA besides electrical stimulation which will cut off the output circuit, blinding of real and sham EA treatment seem difficult and even impossible because of the patients may finally discover their treatment for placebo-control. For clinical trials, blinding is difficult for acupuncture or EA, so real randomised trials seem impossible. To improve compliance of participants, they are allowed to take ibuprofen when necessary and will take 12-session treatment for free. Potential participants will be informed that they will take free-offered ibuprofen to control acute headache attacks before they enrolled in this trial. During the trial, the drug besides ibuprofen will be recorded in the headache diary.

Briefly, the aim of this trial is to confirm the analgesic effect of low- and high-frequency EA and to further explore the dominant frequency of EA for migraine. This study may also confirm the therapeutic difference of low- and high-frequency EA on psychological and emotional changes due to headache attacks.

## Trial status

Participant enrollment will be started on 1 June 2021. The completion is expected to be finished on 31 May 2023 (due to COVID-19). This protocol has been revised four times for version number 4.0, and the date of the latest revision is 13 January 2021.

## Supplementary Information

**Additional file 1.**

## Abbreviations

TCM: Traditional Chinese medicine
EA: Electroacupuncture
WHO: World Health Organization
CNY: China Yuan
USD: United States dollar
GAD: Generalized anxiety disorder
ICHD-3: International Classification of Headache Disorders, 3rd Edition
VAS: Visual analogue scale
SAS: Self-Rating Anxiety Scale
SDS: Self-Rating Depression Scale
MSQ: Migraine Specific Quality of Life Questionnaire
SPSS: Statistical Product and Service Solutions
AE: Adverse event
SAE: Serious adverse event
MITT: Modified intention-to-treat
IQR: Interquartile range
SD: Standard deviation
LOCF: Last observation carried forward
ANOVA: Analysis of variance
ANCOVA: Analysis of covariance
