# Supplementary material for: Comparative effect of electroacupuncture with different frequency on headache attacks in migraine outpatients: study protocol for a randomised placebo-controlled trial
Source: Trials. 2021 Jul 23;22:483. doi: 10.1186/s13063-021-05429-9 (PMC8299449; doi:10.1186/s13063-021-05429-9)
Supplement: Supplementary file 1 — Additional file 1. [file 13063_2021_5429_MOESM1_ESM.pdf]

Supplement data 1: Acupuncture Expectancy Scale

Each of individual may have different expectation for the effects of acupuncture. Please choose the closest answer in the statements to describe your expectation of acupuncture's effect on your illness/symptom before the entire course of acupuncture treatment with "✓".

Statement	Not at All Agree	A Little Agree	Moderately Agree	Mostly Agree	Completely Agree
1.My illness will improve a lot					
2.I will be able to cope with my illness better					
3.The symptoms of my illness will disappear					
4.I will be able to enjoy life more					
5.My mood will improve					
6.My energy level will increase					
7.I will suffer less from this illness					
8.I am confident in the prescribed course of acupuncture treatment(s)					

Supplement data 2: Blinding Evaluation Scale

Each of individual will receive the blinding evaluation at baseline and week 4 post randomisation. Please choose the group you think you are in with "✓".

Timepoint	which group you think you are in
Baseline	☐ 2 Hz EA group<br>☐ 100 Hz EA group<br>☐ Placebo control group<br>☐ I don't know
Week 4 post randomisation	☐ 2 Hz EA group<br>☐ 100 Hz EA group<br>☐ Placebo control group<br>☐ I don't know
